# Including Insonation in Undergraduate Medical School Curriculum

**DOI:** 10.5334/aogh.2472

**Published:** 2019-11-12

**Authors:** Bret P. Nelson, Sukrit Narula, Edgar Argulian, Anjali Bhagra, Jagat Narula

**Affiliations:** 1 Department of Emergency Medicine, Icahn School of Medicine at Mount Sinai, NY, US; 2 Department of Cardiology, Icahn School of Medicine at Mount Sinai, NY, US; 3 Division of General Internal Medicine, Mayo Clinic, Rochester, MN, US

## Abstract

Insonation, or the use of ultrasound, has been proposed to be included in the medical school curriculum, both for education and bedside physical examination. It is important to consider what impact insonation should have on medical student education. Increasingly students are exposed to ultrasound use on clinical rotations, but to what extent should ultrasound be an integrated part of the preclinical curriculum in the United States? Ultrasound can serve to augment an existing curriculum in anatomy, physiology, physical examination, and disease assessment and treatment. In addition, the actual performance and interpretation of the insonation component of physical examination in real time may be an emerging skill set to be expected of medical students. Here we describe the utility and challenges of incorporating an ultrasound curriculum into undergraduate medical education, including examples from institutions that have pioneered this innovative curricular change.

## Introduction

More than a century after the publication of the Flexner Report, curricular reform in medical schools remains a topic of much debate. Crafting a balance between rigorous scientific education and patient-centered care has been a major challenge for schools, and although there have been many reports and guidelines by national organizations there are still numerous curricular models employed in medical schools throughout the United States [1]. One major challenge is the continuous re-appraisal of educational content and techniques in the face of ever-changing technology.

## The case for inclusion of ultrasound in the medical school curriculum

Historically, ultrasound education has been employed during medical school curricula by using large scanners located in imaging laboratories within Radiology, Cardiology, Vascular or Obstetrics departments. With the advent of hand-held scanners it has become possible to scan patients in real time at the point of care, and many specialties such as Emergency Medicine, Surgery, Critical Care plus others have begun to embrace the technology and change their practices [2]. Thus a technology innovation has enabled diverse new groups of users to perform focused sonographic assessment. Addition of ultrasound to four important components of physical examination (i.e. inspection, palpation, percussion, auscultation) has been proposed and has been referred to as *INSONATION*. Point of care insonation is being viewed as a disruptive innovation, changing the paradigm of consultative imaging by specialists to one where imaging may be performed at the bedside by the clinicians directly responsible for the patient care.

A sample of the wide variety of applications for point of care insonation is presented in Table 1. Nearly every specialty has adopted some use of point of care ultrasound to aid diagnosis or guide procedures. While some procedures may be specialty-specific, such as transcranial Doppler or ultrasound-assisted brachytherapy, other indications span through many specialties. Insonation might also be used by non-physicians, such as ultrasound-guided venous access or bladder volume assessments. As clinical practice changes, residency program training requirements follow suit. For example, diagnostic and procedural ultrasound are part of the Model of the Clinical Practice of Emergency Medicine, and the Accreditation Council for Graduate Medical Education (ACGME) explicitly lists ultrasound among the training guidelines for Emergency Medicine, Obstetrics and Gynecology, Ophthalmology, Physical Medicine and Rehabilitation, Psychiatry and Neurology, Radiology and Urology [3]. Therefore, it can be argued that insonation should be introduced in the medical school curriculum as a competency unto itself, in order to better prepare physicians for the skills required in their future careers.

**Table 1:** Insonation is applicable to most medical specialties.

Specialty	Point of Care Insonation

Anesthesia	Guidance for vascular access, regional anesthesia, intraoperative monitoring of fluid status and cardiac function
Cardiology	Echocardiography, intracardiac assessment
Critical care medicine	Procedural guidance, pulmonary assessment, focused echocardiography, hypotension evaluation
Dermatology	Assessment of skin lesions and tumors
Emergency medicine	Trauma assessment, hypotension evaluation, evaluation of ectopic pregnancy, procedural guidance
Endocrinology and endocrine surgery	Assessment of thyroid and parathyroid, procedural guidance
General surgery	Ultrasonography of the breast, procedural guidance, intraoperative assessment
Gynecology	Assessment of cervix, uterus, and adnexa; procedural guidance
Neonatology	Cranial and pulmonary assessments
Nephrology	Vascular access for dialysis
Neurology	Transcranial Doppler, peripheral-nerve evaluation
Obstetrics and maternal–fetal medicine	Assessment of pregnancy, detection of fetal abnormalities, procedural guidance
Ophthalmology	Corneal and retinal assessment
Orthopedic surgery	Musculoskeletal applications
Otolaryngology	Assessment of thyroid, parathyroid, and neck masses; procedural guidance
Pathology	Guidance for fine needle aspiration, biopsy
Pediatrics	Assessment of bladder, procedural guidance
Physical and rehabilitation medicine	Musculoskeletal diagnostic applications, procedure guidance
Pulmonary medicine	Transthoracic pulmonary assessment, endobronchial assessment, procedural guidance
Radiology and interventional radiology	Ultrasonography taken to the patient with interpretation at the bedside, procedural guidance

Adapted from Moore, NEJM 2011.

A study of first-year medical students instructed in insonation demonstrated they were able to detect pathology in 75% of patients with known cardiac disease, where board-certified cardiologists using stethoscopes could detect 49% [4]. Similarly, internal medicine residents employing insonation were able to improve their diagnostic assessment of left ventricle function, valve disease, and left ventricle hypertrophy using ultrasound. Their assessments compared favorably to studies performed by level III echocardiographers, with average sensitivities of 93% and specificities of 99% for major pathology [5]. Insonation during physical examination by medical students and junior residents were found to increase diagnostic accuracy for systolic dysfunction when compared to history and physical examination [6].

The report of the AAMC (Association of American Medical Colleges) and the Howard Hughes Medical Institute (HHMI) committee, *Scientific Foundations for Future Physicians*, stated, “the medical school curriculum should be integrated across disciplines and repeatedly emphasize the importance and relevance of the sciences to medicine” [1]. Understanding of the basic principles of ultrasound is listed in that report as a specific example of a competency to be expected of students entering medical school. But in broader terms ultrasound can layer an understanding of basic scientific principles (anatomy, physiology, etc.) onto clinical scenarios throughout medical training. This layered curriculum has already been implemented in many schools, with integration of ultrasound through pre-clinical and clinical courses. This can help bridge the gap between basic science and clinical care by demonstrating, in real-time, the living anatomy and physiology described in books. It can be used as a “virtual scalpel” to demonstrate anatomy on live subjects without harm. This allows for a three-dimensional assessment of anatomic structures, and the ability to visualize moving structures (such as the heart) in ways not possible with traditional cadaver dissection. Students learning physical examination can use ultrasound to enhance their understating of surface anatomy, organ size and location.

## Challenges

There are several factors which make insonation integration in the curriculum challenging. First, access to hand-held machines is often limited. At most institutions, the larger ultrasound equipment is in continuous clinical use and taking the machines out of the clinical environment can be difficult. In larger medical centers there are enough machines that taking a small number away for a short educational session is feasible. Other institutions have dedicated training machines in use in educational areas or simulation centers. Others have a relationship with industry allowing for educational use of ultrasound equipment for training and research purposes. When large numbers of machines are available it is possible to have large sessions for many medical students simultaneously. Where these options are not feasible, it may be possible to use machines off hours when they are not being used for clinical cases. Smaller sessions with fewer students would also limit the number of machines needed for each session.

Finding time in the curriculum can be difficult, especially when insonation is viewed as an extra skill to add in. Many programs have set up pilot projects where a set amount of time is dedicated to insonation training and practice, or a portion of the existing curriculum is adapted to incorporate insonation as an adjunctive teaching modality. In more progressive and comprehensive curriculum, insonation has been incorporated at the highest level of curricular planning, so the incorporation at multiple levels of medical education has been thought out at a global level.

There is often variability in faculty’s comfort level in teaching insonation, and therefore finding instructors and mentors for the student experience is difficult. While there may be some faculty with a range of ultrasound knowledge sufficient to cover the curriculum requirements, it may be necessary to incorporate faculty development into the curriculum as well. Faculty development in skills as well as teaching styles and learning objectives is an important component of any course, but it is especially important in education given the wide range of comfort levels in using and instructing others in the technology.

Finally, there are some institutions where political pressure may form obstacles to the medical student curriculum in insonation. If some departments feel ownership over the technology there may be reluctance to have a multi-specialty approach towards teaching the curriculum, or the content may be skewed towards a specialty-specific rather than global approach. Educators may even encounter opposition to the concept of an insonation curriculum for medical students if physicians take the stance that insonation is a technology which is not meant for use by many specialties and only graduate level specialized training is appropriate. This political issue is complex, and solutions include open multidisciplinary discussions at the curriculum committee and Dean’s office level to ensure a fair and balanced approach to undergraduate medical education. It is often helpful as well to invoke analogous curricular components which have been used successfully for years. For example, it is quite standard to teach a medical student destined to become a psychiatrist how to use an ophthalmoscope, and an understanding of anatomy on CT scans is commonly expected of Gross Anatomy students. These practices generally meet little resistance from (and pose no threat to) ophthalmologists and radiologists.

## Year 1 Curriculum

Despite major curriculum changes to the preclinical years at many medical schools, the general focus of the first year remains building an understanding of normal anatomy and physiology. To this end Gross Anatomy remains a mainstay of the curriculum, and many different modalities have been described to enhance students’ educational experience. The term “living anatomy” was coined in 1986 as a means of describing anatomy assessments of joint movement, organ examination, etc. by medical students on themselves and each other [7]. Since then the term has been applied to ultrasound assessments of live anatomy as well. The first description of a Gross Anatomy curriculum which included ultrasound was in 1996 in Hannover Medical School in Germany. Since then many other institutions have incorporated this technique [8,9,10,11,12], and insonation can become a disruptive training strategy.

In addition, several institutions in the United States have crafted longitudinal curricula in insonation which begin in the first year and progress throughout the duration of the medical curriculum. In 2006, Wayne State University School of Medicine began a longitudinal curriculum which began with insonation for normal anatomy and basic principles of insonation for procedural skills [13]. The curriculum included hands-on sessions, didactics, clinical correlates, multimedia computer-based content, and faculty mentoring. In the same year the integrated ultrasound curriculum (dubbed iUSC) at the University of South Carolina School of Medicine was begun [14]. The first-year component of this four-year curriculum consisted of ultrasound laboratory sessions and web-based learning (modules online at www.susme.org) as part of the Gross Anatomy curriculum. In addition, the physiology course was augmented by insonation for cardiovascular hemodynamics (cardiac and vascular ultrasound including Doppler assessment). On the other hand, a multi-modal approach was also used to integrate ultrasound into the first-year medical curriculum at University of California, Irvine [15]. Web-based lectures modules (deployed via iTunes at the *UCIMC Ultrasound Education* channel), peer instruction and standardized formative evaluations were used to teach students skills in insonation with image acquisition and interpretation. At the Icahn School of Medicine at Mount Sinai, sessions on insonation for cardiac and vascular systems, and common bedside procedures have been incorporated into the Gross Anatomy Course since 2006 [12]. In addition, the physical examination course is augmented by insonation sessions for the assessment of the heart, gallbladder, aorta, and thorax [19]. Online tutorials and videos (available at www.SinaiEM.us) were incorporated into the course structure, and dedicated faculty development sessions were used to enhance faculty skills. Students at the Ohio State University College of Medicine assess ultrasound anatomy as an adjunct to each body system covered by the Gross Anatomy curriculum. Students may take part in supplemental coursework highlighting ultrasound assessment of hypotension as well [16].

## Year 2 Curriculum

At most schools the second year curriculum focuses on pathology and increasing comfort with physical examination techniques. Several institutions have integrated insonation into this portion of the learning experience as well. At the University of South Carolina School of Medicine ultrasound is incorporated into the physical diagnosis, problem-based learning, and pathophysiology courses. Insonation of organ systems augments physical examination skills, and case-based scenarios require students to incorporate multi-system assessments such as a hypotension evaluation [14]. The Ohio State program continues with a two-week hands-on clinical skill review course, including sessions on ultrasound-guided procedures. An additional elective is available which covers organ assessments in more depth [16]. At Wayne State and the Icahn School of Medicine at Mount Sinai, insonation is incorporated into the physical examination course [17]. Standardized patients are scanned, and image-based case scenarios build on the concepts taught during the physical examination course.

## Year 3–4 Curriculum

In most schools the third and fourth years are devoted to clinical rotations, and the bulk of students’ time is spent in patient care activities. The curriculum assumes increased comfort with normal and abnormal anatomy, and an increased focus on clinical decision-making. Many medical students encounter ultrasound use in their specialty rotations, and dedicated electives focusing on ultrasound have existed within Radiology, Maternal/Fetal Medicine, Cardiology, and Emergency Medicine for years. Five out of six required third-year clerkships at the University of South Carolina School of Medicine incorporate insonation instruction and an insonation-specific competency assessment [14]. During the fourth year, electives in Emergency Medicine insonation, Radiology, and a two-day insonation “Capstone” course are available to interested students.

At Ohio State students complete an adjunct course in insonation image interpretation and indications as part of their core clinical rotations in third year [16]. In addition, fourth-year students undergo more advanced insonation training during their required Emergency Medicine rotation. Interested students may also undertake an advanced ultrasound elective which includes didactic and hands-on sessions, journal club, and participation in Emergency Department or Intensive Care Unit Insonation rounds [18]. The University of California, Irvine established the first fourth-year medical student emergency insonation elective in 2002 [20]. Each two- or four-week rotation includes didactic sessions, image interpretation review, and performance of bedside insonation in the Emergency Department. This format has become common to most Emergency Department-based insonation rotations for students, and the number of programs offering Emergency insonation rotations has grown dramatically in the last decade. Most of the nearly 90 fellowship programs in Emergency insonation offer rotations for medical students.

## Sample Longitudinal Curriculum

Based on the experiences of many medical schools, a sample longitudinal curriculum is included below (Table 2). Educators may consider more or fewer of these components based on resources.

**Table 2:** Sample Longitudinal Insonation Curriculum.

Year	Complementary coursework	Sample Topics

Year 1	Anatomy, physiology	Ultrasound anatomy of cardiovascular system, hepatobiliary system, kidneys, bladder, etc. Dynamic anatomy of heart (cardiac contractility, valve motion), lungs (pleural sliding, diaphragm movement), musculoskeletal system (tendon, join movement, muscular contraction), etc.
Year 2	Pathophysiology, Physical Examination course	Correlation of physical examination and landmark anatomy to ultrasound findings. Cardiac function and relation to hemodynamic assessments such as stroke volume, cardiac output, Starling curve, etc.
Years 3–4	Specialty Rotations	Core topics based on specialty, such as fetal assessments in OB, cardiovascular assessments, bladder volume in Internal Medicine, Thoracic ultrasound in Intensive Care Medicine, Hepatobiliary assessments in Emergency Medicine and Surgery, etc.

## Special Considerations

Since the 1990s there has been a rapid proliferation of online learning modules used in medical education. A recent meta-analysis of over 200 published studies on internet-based instruction found a positive effect for educational outcomes such as knowledge, skills, behaviors and effects on patient care [21]. Given the tight time constraints on medical school curricula in general, many educators have sought to augment traditional classroom time with educational materials which students can access on their own schedule. Given the large amount of content knowledge necessary in insonation, it is not surprising that all medical schools currently training students in insonation incorporate internet-based content delivery to some extent.

The variety of multimedia options used by the different programs reflects the varied needs of each group of students and technology options available. A combination of online tutorials, interactive assessment tools, cases, image archives, and other modalities have been used. The science of asynchronous learning is evolving as adult education theory itself evolves. A recent study investigated which components of internet-based learning were most effective and found interactivity, practice exercises, repetition, and feedback improve learning outcomes [22]. Learner satisfaction was improved with interactivity, online discussion, and audio. In addition to the online modules previously listed, other examples of using interactive online technologies in ultrasound for medical education include the sonographic digital portfolio of saved cases at Ohio State [23], and the use of social media. At Ohio State, ultrasound educators used Twitter to deploy a curriculum of high-yield insonation concepts via “push technology” to followers on the account [24].

Incorporation of insonation in medical education is growing rapidly. In 2011, the University of South Carolina hosted the First World Congress on Ultrasound in Medical Education [25]. Educators and learners from across the globe gathered here to present evidence for educational strategies, collaborate, and engage in hands-on education sessions. Other efforts to increase interaction between learners and teachers include the peer mentoring program and The Ultrasound Challenge at Ohio State [26,27].

## Future directions

The disruptive innovation of portable ultrasound and hence insonation has led to a revolution in medical imaging, empowering nontraditional sonographers outside of Radiology, Cardiology, or Obstetrics to help their patients in real time. As use spreads throughout most medical specialties, machine costs decrease, and more specialty societies create guidelines and training pathways in ultrasound use, medical students will be increasingly exposed to this technology throughout their careers. Moreover, they will be increasingly expected to understand its use when they graduate. We may soon expect many types of clinicians to be imagers as well. While traditional imaging specialties will still retain a high level of proficiency and have access to the most advanced imaging equipment, increasingly other clinicians will use focused examinations to gain immediate and management-changing information about their patients. Thus the novelty of medical student education in sonography will wane, replaced instead by ubiquity.
